# Seasonality and clinical impact of human parainfluenza viruses

**DOI:** 10.1111/irv.12597

**Published:** 2018-08-29

**Authors:** Philip Maykowski, Marie Smithgall, Philip Zachariah, Matthew Oberhardt, Celibell Vargas, Carrie Reed, Ryan T. Demmer, Melissa S. Stockwell, Lisa Saiman

**Affiliations:** ^1^ Department of Epidemiology Mailman School of Public Health Columbia University New York New York; ^2^ Department of Pediatrics Columbia University Medical Center New York New York; ^3^ Department of Pathology Columbia University Medical Center New York New York; ^4^ Value Institute NewYork‐Presbyterian Hospital New York New York; ^5^ Centers for Disease Control and Prevention Atlanta Georgia; ^6^ Department of Population and Family Health Mailman School of Public Health Columbia University New York New York; ^7^ Department of Infection Prevention and Control NewYork‐Presbyterian Hospital New York New York; ^8^ Present address: University of Arizona College of Medicine‐Phoenix Phoenix Arizona; ^9^ Present address: Division of Epidemiology and Community Health School of Public Health University of Minnesota Minneapolis Minnesota

**Keywords:** epidemiology, parainfluenza, respiratory, seasonality, viruses

## Abstract

**Background:**

Widespread availability of rapid diagnostic testing for respiratory viruses allows more in‐depth studies of human parainfluenza viruses (HPIV).

**Objectives:**

This study aimed to assess seasonality of HPIV types 1‐4, clinical outcomes by HPIV type, and risk factors for illness severity.

**Patients/Methods:**

This retrospective study was performed from January 2013 to December 2015 in children and adults with HPIV, detected by multiplex reverse transcription polymerase chain reaction, participating in a community surveillance study of acute respiratory infections (ARIs) in New York City and patients admitted to a tertiary care center in the same neighborhood. Seasonality trends by HPIV type were compared between the community and hospital groups. The associations between HPIV type, demographics, clinical characteristics, and illness severity were assessed.

**Results:**

HPIV was detected in 69 (4%) of 1753 community surveillance participants (median age 9.2 years) and 680 hospitalized patients (median age 6.8 years). Seasonality for HPIV types 1‐3 agreed with previously described patterns; HPIV‐4 occurred annually in late summer and fall. In the community cohort, 22 (32%) participants sought medical care, 9 (13%) reported antibiotic use, and 20 (29%) reported ≥1 day of missed work or school. Among hospitalized patients, 24% had ≥4 chronic conditions. Multivariable ordinal logistic regression demonstrated that increased severity of illness was significantly associated with HPIV‐4 and chronic cardiovascular and respiratory conditions in children and with age ≥65 years and chronic respiratory conditions in adults.

**Conclusions:**

HPIV‐4 presented late summer and early fall annually and was associated with increased severity of illness in hospitalized children.

## 1 INTRODUCTION

Human parainfluenza viruses (HPIV) types 1‐4 are common respiratory pathogens that cause both upper and lower respiratory tract illnesses, especially in young children.^1^ HPIV‐1 and HPIV‐2 are more common in children and more often associated with croup.^1^ HPIV‐3 is more frequently associated with bronchiolitis, bronchitis, and pneumonia. HPIV‐4, while least well‐characterized, has been described as associated with both mild illness in children and lower respiratory tract disease.^2^ HPIV types have varying seasonality, prevalence, and clinical manifestations. In the United States, HPIV‐1 usually occurs in the fall of odd numbered years, HPIV‐2 occurs every fall, and HPIV‐3 occurs in spring and summer.^1^, ^3^ The seasonality of HPIV‐4 has not been as well defined, often due to its low prevalence.^4^ A recent study conducted in Colorado found a year‐round prevalence of HPIV‐4 with peaks in the fall of odd numbered years.^5^ However, estimates of HPIV‐4 prevalence are increasing, potentially due to improved and increased diagnostic testing, but few recent studies have assessed this.^6^, ^7^

Widespread availability of multiplex reverse transcription polymerase chain reaction (RT‐PCR) assays allows for more in‐depth studies of the epidemiology and impact of HPIV types. The population of this study included both a community‐based cohort and hospitalized children and adults with laboratory‐detected HPIV. The inclusion of the community cohort allowed for a broader evaluation of the impact of HPIV than has been previously assessed. The study's objectives were to (a) assess the seasonality of HPIV types 1‐4, (b) determine clinical outcomes by HPIV type, and (c) identify risk factors for increased severity of illness.

## 2 METHODS

### 2.1 Study design, site, and subjects

A retrospective study of participants in a community surveillance cohort and hospitalized patients with HPIV types 1‐4 detected from January 1, 2013 to December 31, 2015 was performed. The community cohort was derived from the Mobile Surveillance for Acute Respiratory Infections (ARIs) and Influenza‐like Illness (ILI) in the Community (MoSAIC) study, a 5‐year community‐based surveillance study in New York City (NYC) that includes 250 households annually.^8^, ^9^ Households were identified by contacting a random sample of participants who had taken part in a large population‐based survey of an urban, primarily immigrant Latino community. For this study, eligible households had 3 or more members with at least 1 member under 18 years of age, were Spanish‐ or English‐speaking, and had a cellular telephone with text messaging.^8^ Text messages were sent twice weekly inquiring about possible respiratory illness among household members. Nasal swabs were collected from ill participants by research staff if at least two of the following were reported to be present: fever/feverishness, runny nose/congestion, sore throat, cough, and/or myalgia. For infants, either runny nose or congestion prompted collection of a swab.

Hospitalized adults and children with laboratory‐detected HPIV admitted to a university‐affiliated medical center in NYC located in the same neighborhood as the community cohort were included. Testing was requested by treating clinicians, and as per the standard of care, is generally recommended for all hospitalizations to evaluate acute respiratory symptoms and guide transmission precautions. Patients hospitalized within 2 calendar‐days of HPIV detection were included. Those with HPIV detected >2 days before admission, >2 days after admission and those with a second positive test for the same HPIV type within 4 weeks of the first positive test were excluded.

### 2.2 Diagnostic testing

Nasopharyngeal swabs for both the community cohort and the hospitalized patients were tested for respiratory pathogens using multiplex RT‐PCR (BioFire Diagnostics, Inc. Salt Lake City, Utah) which has been reported to have 91%‐100% sensitivity and 98%‐100% specificity for HPIV types 1‐4 and detects 20 respiratory pathogens including HPIV, adenovirus, coronavirus (HKU1, NL63, 229E, OC43), human metapneumovirus, rhinovirus/enterovirus, influenza virus (A, A/H1, A/H3, A/H1‐ 2009, B), respiratory syncytial virus, *Mycoplasma*,* Bordetella pertussis*, and *Chlamydophila*.^10^, ^11^ Swabs from the community were tested in a research laboratory at Columbia University Medical Center (CUMC). Swabs from hospitalized patients were tested in the clinical microbiology laboratory at NewYork‐Presbyterian Hospital at CUMC. Blood, urine, and respiratory tract cultures for bacteria were sent by treating clinicians as per the standard of care for suspected infections and also processed by the clinical microbiology laboratory.

### 2.3 Data collection

For the community cohort, days of missed school or work; medical care in primary care clinics, urgent care, or emergency departments; hospitalizations; and use of antibiotics were collected by follow‐up calls at least 15 days after the resolution of respiratory illness.

For hospitalized patients, demographic and clinical characteristics were obtained from electronic medical records (EMR). Primary ICD‐9 diagnoses were categorized as respiratory or nonrespiratory. Patients’ primary and secondary diagnoses were categorized into groups of chronic conditions previously described as risk factors for severe respiratory disease in adults and children with respiratory viruses.^12^, ^13^, ^14^ Conditions were excluded if they could not be classified within a chronic condition category, had <15 occurrences across all patients, or have not been associated with severe respiratory disease, for example bipolar disorder. Manual chart review of readmitted patients was performed to determine whether readmissions within 4 weeks were due to respiratory illness.

Hospital course and healthcare utilization data were obtained from the EMR including radiographs within 2 days of HPIV detection; all antibiotics within 2 days prior to or 7 days after HPIV detection; ICU admission and 30‐day mortality. Respiratory support including use of continuous positive airway pressure (CPAP), mechanical ventilation, or extracorporeal membrane oxygenation (ECMO) was determined by billed procedure codes. Use of bilevel positive airway pressure (BiPAP) or oxygen supplementation was not assessed, as accurate usage data were unavailable in structured electronic sources.

### 2.4 Analysis

To assess seasonality, epidemiologic curves that modeled the detection of each HPIV type in the community cohort versus hospitalized patients were created. Demographic and clinical characteristics of the two groups were analyzed as proportions, means, and/or medians; associations between HPIV type and clinical course were examined using chi‐square, Fisher's exact, and ANOVA tests, as appropriate.

A respiratory severity index was adapted to examine the association of HPIV type with severity of illness.^15^ Three severity categories were created: mild, moderate, and severe. The severe category included patients who died within 30 days of HPIV detection, required ECMO or were mechanically ventilated. Moderate illness included those who were admitted to an ICU or required CPAP which can be provided on non‐ICU floors as well as ICUs. Mild illness included all others.

Putative risk factors for illness severity were examined among hospitalized patients. Bivariate analyses first considered the distribution of illness severity category by levels of possible risk factors. Variables with a significance of *P* < 0.10 in bivariable analysis were included in the multivariable ordinal logistic regression models which were used to regress the odds of illness severity. Initial analyses showed interactions between HPIV type and age likely reflecting heterogeneous populations with different risk factors for severe illness. Thus, different severity models were created for adult and pediatric patients. Multilevel categorical variables were assessed using likelihood ratio tests. The final models were created using a manual backward elimination method, keeping HPIV type included, and dropping the variable with the largest *P*‐value first until all other remaining variables were significant (*P* < 0.05). All statistical analyses were performed in SAS 9.4.^16^

## 3 RESULTS

### 3.1 Subjects

**Figure 1:**
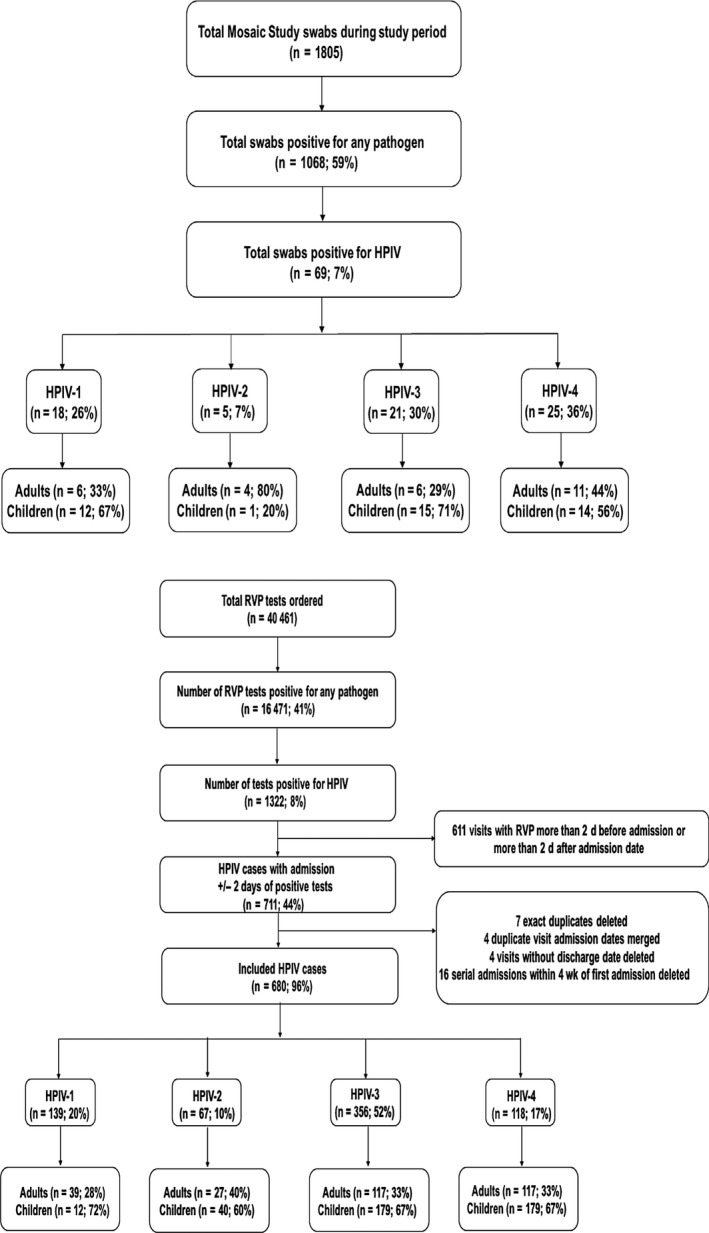
Flowcharts depicting the overall number of respiratory viral panel (RVP) tests ordered which yielded the final number of human parainfluenza virus (HPIV) types in the community cohort (1A) and in hospitalized patients (1B)

In the community cohort, 1805 swabs were obtained, of which 69 (4%) of 1753 participants were positive for at least one HPIV type (Figure 1A). The 69 HPIV‐positive swabs were from 59 households; 10 (14%) households had two individuals with the same HPIV type detected. HPIV‐4 was most common (36.2%). Participants with HPIV detected had a mean and median age of 18.0 (SD: ±18.8) and 9.2 (IQR: 2.82‐30.5) years, respectively; 100% identified as Latino (reflective of the cohort's demographics); and 75% were publicly insured (Table 1). Demographic and clinical characteristics and outcomes were similar among participants with different HPIV types. The most commonly reported symptoms were rhinorrhea (70%) and cough (67%). Twenty‐two (32%) participants reported seeking medical care, nine (15%) reported antibiotic use, none were hospitalized and 20 (29%) reported at least 1 day of missed work or school by participants or caregivers (median 1 [IQR: 1‐2] day per episode in which work or school was missed).

**Table 1:** Comparison of demographic characteristics, symptoms, and outcomes of participants in a community cohort, by human parainfluenza viruses (HPIV) type

Characteristic	HPIV‐1 (n = 18)	HPIV‐2 (n = 5)	HPIV‐3 (n = 21)	HPIV‐4 (n = 25)	Overall (n = 69)	*P*‐value
Sex‐male^a^	8 (44%)	1 (20%)	5 (24%)	14 (56%)	28 (41%)	0.12
Median age, y (Interquartile range)	10.3 (2.9‐31.3)	30.7 (27.8‐36.9)	4.0 (2.1‐28.3)	17.4 (3.2‐29.3)	9.2 (2.8‐30.5)	
Age strata (y)^a^						
<1	0 (0%)	0 (0%)	1 (5%)	3 (12%)	4 (6%)	0.44
1‐4	8 (44%)	1 (20%)	10 (48%)	7 (28%)	26 (38%)	
5‐17	4 (22%)	0 (0%)	4 (19%)	4 (16%)	12 (17%)	
18‐64	6 (33%)	4 (80%)	5 (24%)	11 (44%)	26 (38%)	
≥65	0 (0%)	0 (0%)	1 (5%)	0 (0%)	1 (1%)	
Hispanic ethnicity^a^	18 (100%)	5 (100%)	21 (100%)	25 (100%)	69 (100%)	
Type of insurance^a^						
Commercial	2 (11%)	1 (20%)	4 (19%)	5 (20%)	12 (17%)	0.78
Medicaid	15 (83%)	4 (80%)	14 (67%)	19 (76%)	52 (75%)	
Uninsured	1 (6%)	0 (0%)	3 (14%)	1 (4%)	5 (7%)	
Most common presenting symptoms^a^						
Runny nose	13 (72%)	4 (80%)	14 (67%)	17 (68%)	48 (70%)	0.93
Cough	14 (78%)	4 (80%)	14 (67%)	14 (56%)	46 (67%)	0.44
Sore throat	5 (28%)	0 (0%)	5 (24%)	7 (28%)	21 (30%)	0.10
Fever	5 (28%)	0 (0%)	6 (29%)	4 (16%)	15 (22%)	0.42
Outcomes						
Medically attended^a^	8 (44%)	2 (40%)	4 (19%)	8 (32%)	22 (32%)	0.38
Antibiotic usage^a^	3 (17%)	2 (40%)	2 (10%)	2 (8%)	9 (13%)	0.24
School/work days missed, median [IQR]	1 [1‐1.75]	1.5 [1.25‐1.75]	1 [1‐2]	1 [1‐1]	1 [1‐2]	0.52

HPIV, human parainfluenza virus.

a Column percent of HPIV type.

During the study period, 1607 (4%) of 40 461 multiplex RT‐PCR tests processed in the clinical microbiology laboratory were positive for at least one HPIV type (Figure 1B). Overall, 711 (44%) tests (680 hospitalizations for 663 patients) were positive within 2 days of hospital admission and HPIV‐3 was most common (52.4%). The mean and median age of hospitalized patients were 29.7 (SD: ±33.5) years and 6.8 (IQR: 1.4‐64.3) years, respectively; 45% were white; 38% were Hispanic; and 74% had at least one chronic condition and 24% had ≥4 chronic conditions. Sex, age, and number/type of chronic conditions varied by HPIV type; those with HPIV‐3 were significantly older (Table 2).

**Table 2:** Comparison of demographic and clinical characteristics of hospitalized patients with different human parainfluenza viruses (HPIV)

Characteristic	HPIV‐1 (n = 139)	HPIV‐2 (n = 67)	HPIV‐3 (n = 356)	HPIV‐4 (n = 118)	Overall (n = 680)	*P*‐value
Sex‐male^a^	79 (57%)	42 (63%)	161 (45%)	48 (41%)	328 (48%)	0.006
Median age, y (Interquartile range)	3.1 (1.1‐31.4)	7.2 (1.4‐45.6)	15.9 (1.6‐69.7)	6.5 (1.7‐53.6)	6.8 (1.4‐64.3)	
Age strata (y)^a^						
<1	34 (24%)	11 (16%)	69 (19%)	18 (15%)	132 (19%)	<0.001
1‐<5	55 (40%)	21 (32%)	73 (21%)	37 (31%)	186 (27%)	
5‐<18	11 (8%)	8 (12%)	37 (10%)	16 (14%)	72 (11%)	
18‐<65	17 (12%)	16 (24%)	67 (19%)	26 (22%)	126 (19%)	
≥65	22 (16%)	11 (16%)	110 (31%)	21 (18%)	164 (24%)	
Race^a^						
White	71 (51%)	36 (53%)	151 (42%)	48 (41%)	306 (45%)	0.29
Black	23 (17%)	10 (15%)	56 (16%)	21 (18%)	110 (16%)	
Asian	5 (4%)	1 (1%)	5 (1%)	1 (1%)	12 (2%)	
Other	13 (9%)	5 (7%)	56 (16%)	15 (13%)	89 (13%)	
Unknown	27 (19%)	15 (24%)	88 (25%)	33 (28%)	163 (24%)	
Ethnicity^a^						
Hispanic	51 (37%)	22 (33%)	138 (39%)	45 (38%)	256 (38%)	
Missing	33 (24%)	18 (27%)	103 (29%)	34 (29%)	188 (28%)	
Type of insurance^a^						
Commercial	37 (27%)	16 (24%)	71 (20%)	21 (18%)	145 (21%)	0.002
Medicaid	73 (53%)	34 (50%)	146 (41%)	67 (57%)	320 (47%)	
Medicare	18 (13%)	10 (15%)	110 (31%)	19 (16%)	157 (23%)	
Self‐pay	1 (1%)	1 (1%)	6 (2%)	3 (3%)	11 (2%)	
Unknown	10 (7%)	6 (10%)	23 (6%)	8 (7%)	47 (7%)	
Number of chronic conditions^a^						
0	44 (32%)	21 (32%)	76 (21%)	37 (31%)	178 (26%)	<0.001
1	37 (27%)	16 (24%)	65 (18%)	28 (24%)	146 (21%)	
2	21 (15%)	9 (13%)	42 (12%)	20 (17%)	92 (14%)	
3	14 (10%)	10 (15%)	55 (15%)	22 (19%)	101 (15%)	
≥4	23 (17%)	11 (16%)	118 (33%)	11 (9%)	163 (24%)	
Type of chronic conditions^a^						
Respiratory^b^	46 (33%)	21 (31%)	161 (45%)	51 (43%)	279 (41%)	0.03
Cardiovascular^c^	36 (36%)	23 (34%)	148 (42%)	41 (35%)	248 (36%)	0.01
Immunocompromised^d^	22 (16%)	3 (5%)	76 (21%)	22 (19%)	135 (20%)	0.51
Neurological^e^	18 (22%)	15 (22%)	57 (16%)	17 (14%)	95 (14%)	0.09

HPIV, human parainfluenza virus.

a Column percent of HPIV type.

b Asthma, COPD, and other respiratory disorders.

c Congenital heart disease, hypertension, congestive heart failure, and other cardiovascular disorders.

d HIV, malignancy, transplant recipients, and other immunodeficiency‐associated disorders.

e Epilepsy, Down's Syndrome, dementia, developmental delay, and other neurological disorders.

### 3.2 Seasonality

**Figure 2:**
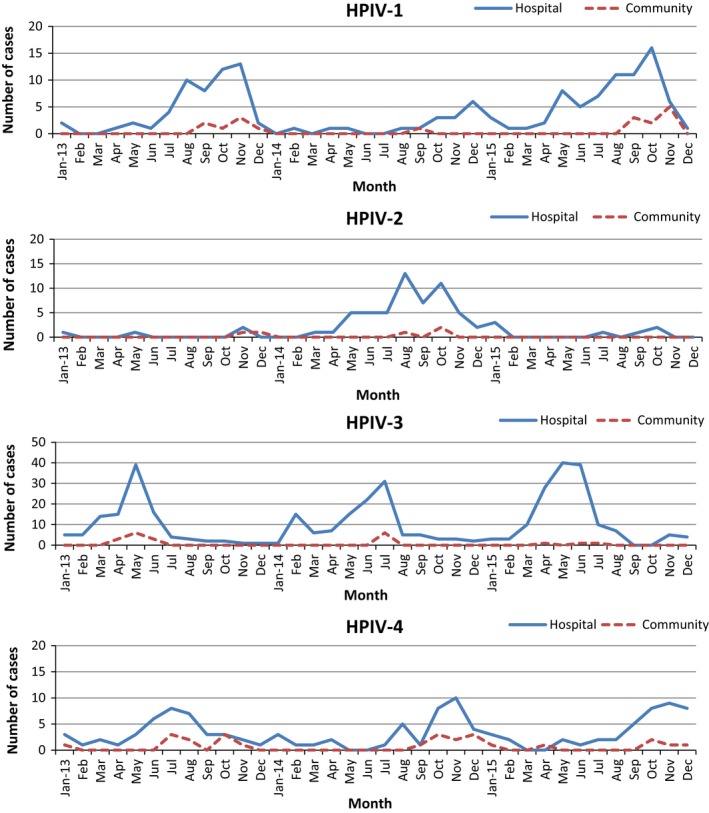
Seasonality of human parainfluenza virus (HPIV) types in community cohort and hospitalized patients. This figure depicts number of human parainfluenza cases (includes co‐infections) detected by month over the study period. Note the *y*‐axis for HPIV‐1, HPIV‐2, and HPIV‐4 includes a smaller maximum value than HPIV‐3 due to differences in their respective prevalence

Seasonality for the four HPIV types was similar in the community cohort and among hospitalized patients (Figure 2). HPIV‐1 largely occurred in the summer and fall of odd numbered years. HPIV‐2 was rarely detected with the exception of the fall of 2014. HPIV‐3 occurred in spring and summer each year. HPIV‐4 occurred following HPIV‐3 each year in late summer through late fall.

### 3.3 Hospital course and co‐infections

The mean length of hospitalization was 5.3 (SD ± 8.4) days and varied by HPIV type (Table 3). The proportion of patients with a respiratory diagnosis varied among HPIV types (highest for HPIV‐4). The proportion of those with chest x‐rays obtained (highest for HPIV‐3) and receiving antibiotics (highest for HPIV‐4) also varied by HPIV type. Overall, 112 (16%) patients were admitted to an ICU, 74 (11%) required CPAP, 29 (4%) were ventilated, and 2 (<1%) required ECMO. Those with HPIV‐4 were more likely to have an ICU admission and had a longer ICU stay than those with other HPIV types. The crude 30‐day mortality rate was 2%. The mean and median age of those who died were 28.4 and 6.8 years, respectively, and only one patient who died had no comorbid conditions. Overall, 118 patients had positive bacterial cultures including 37 blood, 41 urine, and 40 respiratory cultures. The types of viral co‐detections (overall 18%) and of positive bacterial cultures were similar among patients with different HPIV types (Table 3).

**Table 3:** Comparison of hospital course and clinical outcomes of hospitalized patients with different human parainfluenza viruses (HPIV)

Characteristic^a^	HPIV‐1 (n = 139)	HPIV‐2 (n = 67)	HPIV‐3 (n = 356)	HPIV‐4 (n = 118)	Overall (n = 680)	*P*‐value
Primary respiratory diagnosis^a^	98 (71%)	51 (75%)	288 (81%)	100 (85%)	537 (79%)	0.02
Co‐detections	24 (17%)	16 (24%)	64 (18%)	26 (22%)	125 (18%)	0.21
Other HPIV type	0 (0%)	2 (3%)	1 (1%)	2 (1%)	5 (1%)	–
Rhinovirus/enterovirus	16 (15%)	11 (16%)	37 (10%)	15 (13%)	79 (12%)	0.54
Coronaviruses	0 (0%)	1 (1%)	11 (3%)	4 (3%)	16 (2%)	0.18
Respiratory syncytial virus	3 (2%)	2 (3%)	4 (1%)	4 (3%)	13 (2%)	0.39
Adenovirus	0 (0%)	1 (1%)	7 (2%)	1 (1%)	9 (1%)	0.36
Influenza A	0 (0%)	2 (3%)	4 (1%)	0 (0%)	6 (1%)	0.12
Influenza B	0 (0%)	0 (0%)	2 (1%)	0 (0%)	2 (<1%)	0.61
Other^b^	0 (0%)	2 (3%)	4 (1%)	2 (1%)	8 (1%)	–
Positive Bacterial Culture	24 (17%)	14 (21%)	68 (19%)	12 (10%)	118 (17%)	0.13
Blood	9 (6%)	4 (6%)	19 (5%)	5 (4%)	37 (5%)	0.88
Urine	9 (6%)	5 (7%)	27 (8%)	2 (1%)	43 (6%)	0.81
Respiratory	6 (4%)	5 (7%)	22 (6%)	5 (4%)	38 (6%)	0.09
Radiographs obtained	83 (60%)	45 (66%)	266 (75%)	84 (71%)	478 (70%)	0.01
Antibiotics received	82 (59%)	38 (56%)	262 (74%)	118 (100%)	500 (73%)	<0.001
Outcomes						
Median length of stay, days (IQR)	2 (1‐4)	2 (1‐4)	3 (2‐6)	3 (2‐7)	3 (2‐6)	0.01
ICU admission	15 (11%)	8 (12%)	58 (16%)	31 (26%)	112 (16%)	0.006
Mean ICU hours	10.7 (±44.7)	7.22 (±24.7)	29.9 (±112.3)	38.4 (±94.7)	25.2 (±93.3)	0.027
Continuous positive airway pressure	14 (10%)	5 (7%)	38 (11%)	17 (14%)	74 (11%)	0.49
Mechanical ventilation	5 (4%)	2 (3%)	17 (5%)	5 (4%)	29 (4%)	0.89
Extracorporeal membrane oxygenation	0 (0%)	0 (0%)	2 (1%)	0 (0%)	2 (<1%)	–
Respiratory readmission	1 (1%)	0 (0%)	7 (2%)	0 (0%)	8 (1%)	0.23
Crude in‐hospital mortality	3 (2%)	1 (2%)	6 (2%)	2 (2%)	12 (2%)	0.98

HPIV, human parainfluenza virus.

a Column percent of HPIV type.

b Includes: Human metapneumovirus (n = 6) and pertussis (n = 2).

### 3.4 Severity of illness in hospitalized patients

Among all hospitalized patients with HPIV, 35 (5%) had severe, 124 (18%) had moderate, and 521 (77%) had mild illnesses. Among the 390 children, 23 (6%) had severe, 83 (21%) had moderate, and 162 (73%) had mild illness (Table 4). In the final adjusted model for children, HPIV‐4, and respiratory and cardiovascular conditions were associated with higher odds of increased severity of illness. Among the 290 adults, 12 (4%) had severe, 41 (14%) had moderate, and 237 (82%) had mild illness (Table 5). In the final adjusted model for adults, age ≥65 years and respiratory conditions were associated with higher odds of increased severity of illness.

**Table 4:** Risk factors for increased severity of illness in children with different human parainfluenza viruses (HPIV)

Variable^a^	Severity^a^			Bivariate *P*‐value	Multivariable aOR (CI_95_)^g^
	Mild (n = 284)	Moderate (n = 83)	Severe (n = 23)		
Sex—male	162 (73)	47 (21)	12 (5)	0.77	–
Age (in years)^b^	–	–	–	0.12	–
Race^a^					
White (ref)	149 (74)	48 (24)	5 (2)	0.01	–
Black	46 (69)	15 (22)	6 (9)		
Asian	3 (38)	1 (13)	4 (50)		
Other	32 (76)	6 (14)	4 (10)		
Missing	54 (76)	13 (18)	4 (6)		
Insurance^a^					
Commercial (ref)	80 (70)	30 (26)	5 (4)	0.83	–
Medicaid	181 (74)	46 (19)	16 (7)		
Self‐pay	5 (83)	0 (0)	1 (17)		
Unknown	18 (69)	7 (27)	1 (4)		
HPIV type^a^					
1 (ref)	84 (84)	12 (12)	4 (4)	–	
2	31 (78)	7 (18)	2 (5)	0.3693	1.60 (0.63, 4.10)
3	130 (73)	36 (20)	13 (7)	0.03	1.73 (0.91, 3.30)
4	39 (55)	28 (39)	4 (6)	<0.001	3.85 (1.87, 7.94)
Number of chronic conditions^b^					
0	119 (81)	25 (17)	3 (2)	<0.001	
1	90 (76)	25 (21)	4 (4)		
2	36 (69)	11 (21)	5 (10)		
3	23 (59)	11 (28)	5 (13)		
≥4	16 (48)	11 (33)	6 (18)		
Type of chronic conditions^a^					
Respiratory^c^	80 (62)	38 (29)	12 (9)	<0.001	2.25 (1.37, 3.70)
Cardiovascular^d^	32 (47)	26 (38)	10 (15)	<0.001	3.81 (2.21, 6.55)
Immunocompromised^e^	26 (64)	12 (29)	3 (7)	0.17	
Neurological^f^	32 (57)	17 (30)	7 (13)	0.003	
Co‐infection^a^	75 (76)	19 (19)	5 (5)	0.44	

HPIV, human parainfluenza virus.

a Row percent of variable.

b Modeled continuously.

c Asthma, chronic obstructive pulmonary disease, pulmonary fibrosis, and other respiratory disorders.

d Congenital heart disease, hypertension, congestive heart failure, and other cardiovascular disorders.

e HIV, malignancy, transplant recipients, and other immunodeficiency‐associated disorders.

f Epilepsy, Down's Syndrome.

g Final model created using a manual backward elimination method.

**Table 5:** Risk factors for increased severity of illness in adults with different human parainfluenza viruses (HPIV)

Variable	Severity^a^			Bivariate *P*‐value	Multivariable aOR (CI_95_)^g^
	Mild (n = 237)	Moderate (n = 41)	Severe (n = 12)		
Sex—male	85 (79)	17 (16)	5 (5)	0.44	–
Age (in y)					
18‐64 (ref)	109 (87)	14 (11)	3 (2)	–	
≥65	128 (78)	27 (16)	9 (6)	0.06	2.03 (1.01‐4.11)
Race					
White (ref)	88 (85)	12 (11)	4 (4)	0.63	–
Black	36 (84)	6 (14)	1 (2)		
Asian	3 (75)	1 (25)	0 (0)		
Other	37 (79)	8 (17)	2 (4)		
Missing	73 (79)	14 (15)	5 (6)		
Insurance					
Private (ref)	27 (90)	2 (7)	1 (3)	0.83	–
Medicaid	65 (84)	9 (12)	3 (4)		
Medicare	121 (77)	28 (18)	8 (5)		
Self‐pay	4 (80)	1 (20)	0 (0)		
Unknown	20 (95)	1 (5)	0 (0)		
HPIV Type					
1 (ref)	28 (72)	9 (23)	2 (5)	–	–
2	24 (89)	3 (11)	0 (0)	0.37	0.35 (0.08‐1.44)
3	144 (81)	24 (14)	9 (5)	0.03	0.53 (0.24‐1.20)
4	41 (87)	5 (11)	1 (2)	<0.001	0.36 (0.12‐1.10)
Number of chronic conditions^b^					
0	17 (94)	1 (6)	0 (0)	<0.001	–
1	23 (85)	4 (14)	0 (0)		
2	36 (90)	2 (5)	2 (5)		
3	53 (85)	6 (10)	3 (5)		
≥4	108 (76)	28 (20)	7 (5)		
Type of chronic conditions					
Respiratory^c^	113 (76)	27 (18)	9 (6)	<0.001	2.51 (1.3‐4.79)
Cardiovascular^d^	146 (81)	29 (16)	5 (3)	<0.001	0.73 (0.37‐1.46)
Immunocompromised^e^	81 (86)	11 (12)	2 (2)	0.17	–
Neurological^f^	29 (75)	8 (20)	2 (5)	0.003	–
Co‐infection	20 (77)	4 (15)	2 (8)	0.44	–

HPIV, human parainfluenza virus.

a Number (row percentage).

b Modeled continuously.

c Asthma, chronic obstructive pulmonary disease, pulmonary fibrosis, and other respiratory disorders.

d Congenital heart disease, hypertension, congestive heart failure, and other cardiovascular disorders.

e HIV, malignancy, transplant recipients, and other immunodeficiency‐associated disorders.

f Epilepsy, Down's Syndrome.

g Final model created using a manual backward elimination method.

## 4 DISCUSSION

We had a unique opportunity to compare the epidemiology and clinical impact of HPIV types among individuals in a community‐based surveillance study and among hospitalized patients during the same time and in the same geographic area. The seasonality of HPIV types 1‐3 was consistent with published trends,^1^, ^3^ but we found that HPIV‐4 occurred annually in late summer and fall, suggestive of a local epidemiologic trend that has not been previously described. We found that HPIV‐3 was most common among hospitalized patients (52%) while HPIV‐4 was most common in the community cohort (36%). The relative frequency of HPIV‐4 has varied depending on the population studied; among hospitalized Chinese children under 5 years of age^17^ and among children hospitalized in Colorado,^5^ it was the second most common HPIV type (18% vs 31%, respectively) while among children ≤24 months attending day care, second only to HPIV‐3, 10% of HPIV illnesses were due to HPIV‐4.^18^

In the community cohort of the current study, demographic, and clinical characteristics, and outcomes associated with different HPIV types were similar. HPIV‐associated illnesses contributed to healthcare burden and cost as 32% of ARI episodes were medically attended, 9% received antibiotics, and 29% missed work or school. Few studies have examined HPIV in a community‐based cohort, but among infants in daycare, the clinical presentations and severity of illness were also similar with different HPIV types.^18^

Demographic and clinical characteristics, and outcomes did vary by HPIV type in hospitalized patients. Those with HPIV‐3 had longer hospitalizations, were more likely to be ≥65 years old, and have ≥4 chronic conditions. They were also more likely to have chest x‐rays performed, potentially reflective of their underlying conditions. In contrast, those with HPIV‐4 all received antibiotics and were more likely to have an ICU admission with longer ICU stays. Those with HPIV‐4 were most likely to have a primary respiratory diagnosis, supporting the pathogenicity of HPIV‐4 as previously reported.^19^, ^20^

We modified a previously validated severity of illness score to further explore the predictors of illness severity. In children, increased severity of illness was associated with respiratory conditions, cardiovascular conditions and HPIV‐4 while in adults, increased severity of illness was associated with age ≥65 years and cardiovascular conditions. The association of comorbid conditions and severe illness in those with HPIV is consistent with other respiratory viruses, most notably RSV and influenza, which both can exacerbate underlying cardiac and pulmonary conditions.^12^, ^13^, ^14^, ^21^, ^22^ This may also explain why 21% of hospitalized patients had a nonrespiratory primary diagnosis.

The rate of co‐detection was similar across HPIV strains and as others have shown, viral co‐detections did not impact severity of disease.^23^ However, due to limited sample size, we could not assess the impact of specific co‐infections. While only 17% of HPIV‐positive patients had a positive bacterial culture, 73% received at least one dose of an antibiotic between 2 days before and 7 days after HPIV detection. Thus, most antibiotic usage represented empiric therapy, suggesting an opportunity for antibiotic stewardship.

This study had limitations. The community cohort was 100% Hispanic/Latino, reducing the study's generalizability, and small, limiting our ability to find differences in severity of illness or symptomatology associated with HPIV types. Our hospital is a referral center and cares for many patients with underlying conditions. Furthermore, its referral status could impact accurate interpretation of “local” epidemiology as only 32% of the hospitalized patients lived in the same zip codes as the MoSAIC study participants (M. Stockwell, personal communication). Testing of hospitalized patients relied on clinicians’ judgement. We did not review radiographic findings or capture BiPAP use which may have biased assessment of severity of illness. The severity of illness score was developed for children and not previously tested in adults. Use of administrative data restricted our ability to assess patients’ symptomatology. Although most patients had a respiratory diagnosis, we may have misclassified HPIV infections that actually represented prolonged HPIV shedding from previous illnesses. We did not classify positive bacterial cultures as consistent with infection vs. colonization vs. contamination.

## 5 CONCLUSIONS

In this study, each HPIV type demonstrated seasonality, but each was similar in the community and among hospitalized patients. We found that HPIV‐4 had distinct epidemiology, not previously described, and was associated with increased severity of illness in hospitalized children. As demonstrated for other respiratory viruses, older age and underlying conditions, particularly respiratory and cardiac conditions, were associated with increased severity of illness. Additional studies exploring HPIV incidence and severity in both children and adults could help reinforce the need for vaccine development.

## CONFLICT OF INTEREST

None.
